# Emerging trends in clinical cancer genomic research

**DOI:** 10.20892/j.issn.2095-3941.2023.0383

**Published:** 2023-11-28

**Authors:** Yingyan Yu

**Affiliations:** Department of General Surgery of Ruijin Hospital, Shanghai Institute of Digestive Surgery, and Shanghai Key Laboratory for Gastric Neoplasms, Shanghai Jiao Tong University School of Medicine, Shanghai 200025, China

Academic activities are resuming now that the global COVID-19 pandemic has subsided. On 14-17 June 2023 the 15^th^ International Gastric Cancer Congress (IGCC2023) of the International Gastric Cancer Association was held in Yokohama, Japan. More than 1300 experts from 44 countries discussed the hot issues pertaining to stomach cancer. Greater than 800 abstracts were submitted for presentation at the conference. In the past 3 years the pace of scientific research has slowed, but not stopped. The author has noticed that “omics”-based research involving gastric cancer is closely following the leading edge of human genome research worldwide. Specifically, gastric research have focused on the following cancer genome research trends: from a single reference genome to the reference pangenome; from single population to cross-race; from cancer cell-centered to microenvironment combined; and from next-generation sequencing (NGS) with short reads to third-generation sequencing with long reads. Herein the author summarizes emerging trends in gastric cancer research combined with new progress in the field of solid cancer research.

## Trend one: from single reference genome- to reference pangenome-based methodology

With the increasing amount of human whole genome sequencing (WGS) data, scientists have realized that the human reference genome (the current version is GRCh38) does not reflect global genetic diversity^1^; however, GRCh38 is generally considered the standard when we search the genetic variation of the cancer genome, resulting in inevitable loss of some important variations from a given population. Therefore, it is challenging to determine disease heterogeneity based on a single reference-based cancer genomic study. Scientists have attempted to explore pangenome-based human genome research. For example, on 11 May and 14 June 2023, the internationally renowned journal, *Nature*, published a series of papers on human pangenomic studies, including the first draft of the human pangenome constructed by the Human Pangenome Reference Consortium (HPRC)^2^, and the pangenome reference covering the whole genome sequences from 36 Chinese minorities constructed by the Chinese Pangenome Consortium (CPC)^3^. Yu and Zhu (Ruijin Hospital Affiliated to Shanghai Jiao Tong University School of Medicine, Shanghai, China) proposed the first pangenomic characteristics of Chinese gastric cancer in the Genomics Section at the IGCC2023. As is well-known, gastric cancer seriously endangers the health of people in East Asian countries, including China. Whether a distinct ethnic tendency is related to specific genomic variations has not been determined. Although the results of years of research have shown that infection with *Helicobacter pylori (Hp)* or Epstein-Barr virus (EBV), and Oriental dietary habits are associated with the development of gastric cancer, little is known about the role of genomic variants on the development of gastric cancer. A pangenomic analysis using WGS data from tumor and healthy control tissues of 185 Han Chinese with gastric cancer revealed that 80.88 Mb that did not map to the human reference genome (GRCh38). By matching genomic data from each individual to the pangenome constructed from GRCh38 plus unmapped sequences, an abnormal absence variation of a gene group (*GSTM1*, *ACOT1*, *SIGLEC14*, and *UGT2B17*) was identified. Based on a comparison of WGS data from different ethnic groups in the public database, the absence frequency (41%–71%) of these 4 genes in the gastric cancer population was much higher than reported in European and American descendants (4.6%–46%)^4^. The “presence” or “absence” variation of genes reveals the possible genetic basis for the higher incidence of gastric cancer in Chinese and East Asians. This new finding provides a directional reference for subsequent genetic intervention treatment and clinical medication.

## Trend two: cancer genome research from a single population to cross-race

Aburatani and co-workers^5^ (University of Tokyo, Tokyo, Japan) conducted a genomic study involving cross-race for gastric cancer. The study included 1335 WGS gastric cancer data from multi-ancestry patients. The Asian patients were from China, South Korea, Singapore, and Vietnam. By mapping to a single reference genome, 77 significantly mutant genes were identified, including *ARHGAP5* and *TRIM49C*. Based on correlation analysis with histology, *PIGR* and *SOX9* mutations were identified as driver genes of diffuse-type gastric cancer. The mutation caused by EBV infection was shown to be related to patient ethnicity. Copy number variation was less common in gastric cancer tissues caused by EBV infection, while gene mutations involving the *PI3K*/*MAPK* pathway, as well as gene amplification of *PD-L1*, *PD-L2*, and *JAK2*, were more common. EBV can activate *CTNNB1*. In addition, based on mutation analysis of patients from different races, the *MDM2*, *CDKN2A*, *ARID2*, *PTEN*, and *MUC6* mutations were more common in European patients, while the *TP53* mutation was more common in East Asian patients. A total of 26 significantly mutated genes, including *ARID1A*, *ACVR2A*, *B2M*, *RNF43*, *PTEN*, and *HLA-B*, were identified in gastric cancer with a higher tumor mutation burden (TMB). Although copy number amplification in hypermutant gastric cancer was not a major change, *CD274* (*PD-L1*) amplification was detected in 2.8% of cases.

Japanese scientists reported one distinct gastric cancer subclass in Asian patients with a clear alcohol-associated mutation signature and strong Asian specificity, which was attributable to alcohol intake behavior, smoking habit, and an Asian-specific defective *ALDH2* allele in most cases. The Japanese scientists noticed that alcohol-related gastric cancers have a lower TMB. In addition, the Japanese scientists found frequent (7.4%) germline *CDH1* variants among Japanese gastric cancers, most of which were attributed to a few recurrent single nucleotide variants shared by Japanese and Koreans, suggesting the existence of common ancestral events among East Asians. Approximately one-fifth of diffuse-type gastric cancers in the Lauren classification were attributable to a combination of alcohol intake and a defective *ALDH2* allele or *CDH1* variants. Therefore, the double impact of germline variants (*ALDH2* or *CDH1*) and lifestyles are attributed to gastric carcinogenesis of East Asians in high incidence areas^6^. The results facilitated the trans-ethnic characterization of somatic and germline genetics of gastric cancers. An *ALDH2* gene deficiency was also identified in the Chinese population. *ALDH2* is a member of the aldehyde dehydrogenase superfamily and is involved in the metabolic processing of aldehydes. *ALDH2* has a cytoprotective role in gastric mucosa cells by removing aldehydes produced during normal metabolism. Our previous study showed that the cytoprotective role of *ALDH2* is mediated by metabolism of 4-hydroxynonenal (4-HNE), an oxidative stress-induced lipid peroxidation agent. An increased level of 4-HNE contributes to the DNA damage^7^. Recently, Mezghani and co-workers^8^ reported that molecular subtype-associated biomarkers of head and neck cancer are different in African and European ancestries. Higher frequencies of *TP53*, *MYO18B*, *KMT2D*, and *UNC13C* mutations and a lower frequency of *PIK3CA* mutations were observed in black patients. A significantly increased expression of *c-MYC* of tumor was observed in black patients, which was associated with poor outcomes^8^. The cross-race studies provide a potential clinical significance for more targeted or individualized diagnosis, and treatment for improving health outcomes.

## Trend three: from cancer cell-centered to microenvironment-combined

Peritoneal metastasis is one of the most common metastatic sites in patients with advanced gastric cancer; the prognosis is dismal. With the popularization and wide application of diagnostic laparoscopy in disease staging, studies involving peritoneal metastatic cancer have increased. Gastric cancer with peritoneal metastasis tends to be resistant to traditional chemotherapy. Advances in genomic sequencing and molecular profiling have revealed several promising therapeutic targets, particularly with respect to the role of the tumor microenvironment in the peritoneum. Tan et al.^9^ (National University of Singapore, Queenstown, Singapore) reviewed the new knowledge on variation characteristics of the peritoneal microenvironment in patients with gastric cancer. Based on newly identified molecular targets, several translational studies and clinical trials have been designed and carried out. The peritoneum consists of a basement membrane, mesothelial cells, and connective tissue. Tan et al.^9^ divided peritoneal metastasis-associated factors into four categories, including cancer-related, peritoneal microenvironment, paracrine, and biomechanical forces factors. Cancer-related factors include genomic variation, epithelial-mesenchymal transition (EMT), and Hippo pathway activation. Peritoneal microenvironment factors include mesothelial-mesenchymal transition, immune cells, and angiogenesis. Paracrine factors involve cytokine, chemokine, and growth factors. Biomechanical forces factors (physical factors) include fluid and solid pressure. The concept of mesothelial-mesenchymal transition was introduced, and refers to the observation that after cancer cells adhere to mesothelial cells and sub-mesothelial connective tissues integrins, the mesothelial cells secrete adhesion molecules and gradually acquire the features of cancer-associated fibroblasts (CAFs). CAFs activate the TGF-β signaling pathway and promote cancer proliferation. In addition, CAFs secrete interleukins and other growth factors. With respect to the immunosuppressive niche, few studies have been conducted involving immune cells within the peritoneal metastasis. One RNA-seq study of the peritoneal microenvironment showed that TIM-3, galectin-9, and VISTA immune checkpoints are highly expressed when the peritoneum is in an immunosuppressive microenvironment. The omental neutrophils have been shown to generate extracellular traps, involving the release of a protein-rich chromatin web that promotes the progression of peritoneal metastasis. In addition, peritoneal resident macrophages can express higher levels of Tim-4, thereby inhibiting the antitumor activity of CD8 T cells. Therefore, blocking Tim-4 may become a new strategy for the treatment of peritoneal metastasis. In the peritoneal cavity, both cancer and mesothelial cells secrete VEGF and PDGF, which promote abnormal neovascularization with increased permeability^9^.

In addition, Yoo and co-workers (Seoul National University, Seoul, South Korea) reported use of The Cancer Genome Atlas (TCGA) database to explore factors related to vascular metastasis in gastric cancer. The study focused on genes related to hematogenous metastasis. Yoo et al. identified 95 mutated genes associated with vascular metastasis and divided gene expression profiles from 258 cases into the following 4 groups based on the presence or absence of vascular metastasis: non-metastatic group (N); hematogenous metastasis group (H); regional metastasis group (L); and distal metastasis group (D). Yoo et al. compared RNA-seq data between the four groups and found seven significant difference genes. Compared to the H group, the expression of *ARIDA1* increased by 1.29 times (*P* = 0.018), *ERBB3* increased by 1.44 times (*P* = 0.036), and *FGFR3* increased by 2.82 times (*P* = 0.049) in group L. Compared to group H, the expression of *BAP1* increased by 1.34 times (*P* = 0.001), *TSC2* increased by 1.29 times (*P* = 0.012), and *KDR* increased by 1.57 times (*P* = 0.018) in group D. The researchers are currently verifying gene functions related to hematogenous metastasis of gastric cancer.

Accumulating evidence shows that immune system aging leads to impairment of innate and adaptive immune processes, which can create an inflammatory environment; however, the role of immune-related pathways in aging and cancer remains elusive. Wang and colleagues investigated immune-related genes and pathways among 25 cancer types using genomic and transcriptomic data from TCGA and Genotype-Tissue Expression (GTEx) databases, and found several immune- and aging-related genes in pan-cancer. These genes, in turn, were utilized for potential immunotherapy drug discovery. The potential drug targets include the *FYN*, *JUN*, and *SRC* genes. The work of Wang and colleagues highlight the importance of immune-related genes and pathways with oncogenic roles in the aging process^10^.

## Trend four: genomic sequencing using the short-to-long read method

NGS techniques, which are more focused on capturing mutations, have revolutionized clinical practice. Short-read DNA sequencing technology has been combined in novel ways with other multi-omic approaches to gain unprecedented biological insight into disease. These technologies have improved disease diagnosis, facilitating investigations of premalignant lesions, such as intestinal metaplasia^11^. Despite advances in NGS, structural variants (SVs) are difficult to detect due to the short-read length (usually 100–250 base pair), while long reads (10–20 kb) with third-generation sequencing may improve SV detection. The SVs include large insertions and deletions (at least 50 bp in length), inversions, duplications, translocations, and complex combinations of these mutations^12^.

Pacific Biosciences (PacBio; San Diego, CA, USA) developed single-molecule real-time (SMRT) sequencing. SMRT sequencing requires 10 μg of DNA as the input, which sometimes imposes a burden when only a limited amount of DNA is available in small cancers or early-stage cancers. Nanopore-type sequencers (MinION and PromethION) have been developed by Oxford Nanopore Technologies (ONT; Science Park, Oxford, UK). Generally, it is thought that an ONT sequencer can produce longer reads than a PacBio sequencer. In addition, the input DNA requirement is lower for the ONT sequencer than the PacBio sequencer, although approximately 1 μg of DNA is still required^13^.

Huang and co-workers^14^ compared the transcriptome sequenced using the PacBio long-read method and Illumina short-read method for 10 gastric cancer cell lines covering 4 major molecular subtypes (chromosomal-unstable, EBV-positive, genome-stable, and microsatellite-unstable). Using the long-read method, Hung and co-workers identified 60,239 non-redundant full-length transcripts, of which 66.8% were novel compared to the short-read method. Novel isoforms are more likely to be subtype-specific and expressed at lower levels with a larger number of exons or with longer isoform/coding sequence lengths. Huang and co-workers further identified several cancer-associated isoforms, including novel variants of oncogenes. The results are a rich resource for long-read transcriptome analysis with deeper insight into malignancies. Currently, genome-wide variation studies using third-generation sequencing technology for liver (Oxford nanopore)^15^, pancreatic (Oxford nanopore)^16^, and colorectal cancers (Oxford nanopore)^17^ have been published, but reports for gastric cancers are lacking. It is believed that relevant results will be produced in the near future.

## Conclusions

Although cancer genomic research is consistent with the trend in human genomic research, pangenome- and long-read sequencing-based studies are limited, which may be due to the complex analytic technologies and the higher demand for computing resources on the large amounts of data processing^18,19^. In addition, the requirement for high-quality biological samples of third-generation sequencing technologies may limit widespread clinical use.

## References

[1] Yang X, Lee WP, Ye K, Lee C (2019). One reference genome is not enough. Genome Biol.

[2] Liao WW, Asri M, Ebler J, Doerr D, Haukness M, Hickey G (2023). A draft human pangenome reference. Nature.

[3] Gao Y, Yang X, Chen H, Tan X, Yang Z, Deng L (2023). A pangenome reference of 36 Chinese populations. Nature.

[4] Yu Y, Zhang Z, Dong X, Yang R, Duan Z, Xiang Z (2022). Pangenomic analysis of Chinese gastric cancer. Nat Commun.

[5] Totoki Y, Saito-Adachi M, Shiraishi Y, Komura D, Nakamura H, Suzuki A (2023). Multiancestry genomic and transcriptomic analysis of gastric cancer. Nat Genet.

[6] Suzuki A, Katoh H, Komura D, Kakiuchi M, Tagashira A, Yamamoto S (2020). Defined lifestyle and germline factors predispose Asian populations to gastric cancer. Sci Adv.

[7] Duan Y, Gao Y, Zhang J, Chen Y, Jiang Y, Ji J (2016). Mitochondrial aldehyde dehydrogenase 2 protects gastric mucosa cells against DNA damage caused by oxidative stress. Free Radic Biol Med.

[8] Mezghani N, Yao A, Vasilyeva D, Kaplan N, Shackelford A, Yoon A (2023). Molecular subtypes of head and neck cancer in patients of African ancestry. Clin Cancer Res.

[9] Gwee YX, Chia DKA, So J, Ceelen W, Yong WP, Tan P (2022). Integration of genomic biology into therapeutic strategies of gastric cancer peritoneal metastasis. J Clin Oncol.

[10] Wang X, Guo S, Zhou H, Sun Y, Gan J, Zhang Y (2023). Immune pathways with aging characteristics improve immunotherapy benefits and drug prediction in human cancer. Cancers (Basel).

[11] Yoshida T, Yatabe Y, Kato K, Ishii G, Hamada A, Mano H (2023). The evolution of cancer genomic medicine in Japan and the role of the National Cancer Center Japan. Cancer Biol Med.

[12] Sakamoto Y, Zaha S, Suzuki Y, Seki M, Suzuki A (2021). Application of long-read sequencing to the detection of structural variants in human cancer genomes. Comput Struct Biotechnol J.

[13] Athanasopoulou K, Boti MA, Adamopoulos PG, Skourou PC, Scorilas A (2021). Third-generation sequencing: the spearhead towards the radical transformation of modern genomics. Life (Basel).

[14] Huang KK, Huang J, Wu JKL, Lee M, Tay ST, Kumar V (2021). Long-read transcriptome sequencing reveals abundant promoter diversity in distinct molecular subtypes of gastric cancer. Genome Biol.

[15] Fujimoto A, Wong JH, Yoshii Y, Akiyama S, Tanaka A, Yagi H (2021). Whole-genome sequencing with long reads reveals complex structure and origin of structural variation in human genetic variations and somatic mutations in cancer. Genome Med.

[16] Zhou D, Guo S, Wang Y, Zhao J, Liu H, Zhou F (2023). Functional characteristics of DNA N6-methyladenine modification based on long-read sequencing in pancreatic cancer. Brief Funct Genomics.

[17] Xu L, Wang X, Lu X, Liang F, Liu Z, Zhang H (2023). Long-read sequencing identifies novel structural variations in colorectal cancer. PLoS Genet.

[18] Yu Y, Wei C (2020). A powerful HUPAN on a pan-genome study: significance and perspectives. Cancer Biol Med.

[19] Duan Z, Qiao Y, Lu J, Lu H, Zhang W, Yan F (2019). HUPAN: a pan-genome analysis pipeline for human genomes. Genome Biol.

